# Synthesis and Properties of the Helium Clathrate and Defect Perovskite [He_2–*x*_□_*x*_][CaNb]F_6_

**DOI:** 10.1021/acs.jpcc.4c02174

**Published:** 2024-06-21

**Authors:** Shangye Ma, Brett R. Hester, Anthony J. Lloyd, Antonio M. dos Santos, Jamie J. Molaison, Angus P. Wilkinson

**Affiliations:** † School of Chemistry and Biochemistry, Georgia Institute of Technology, Atlanta, Georgia 30332-0400, United States; ‡ Neutron Scattering Division, Oak Ridge National Laboratory, Oak Ridge, Tennessee 37831, United States; § School of Materials Science and Engineering, Georgia Institute of Technology, Atlanta, Georgia 30332-0245, United States

## Abstract

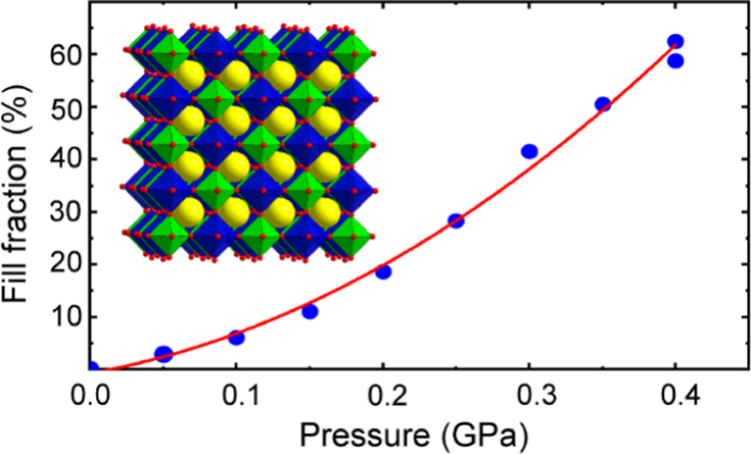

The defect double perovskite [He_2–*x*_□_*x*_][CaNb]F_6_, with helium on its A-site, can be prepared by the insertion of helium into ReO_3_-type CaNbF_6_ at high pressure. Upon cooling from 300 to 100 K under 0.4 GPa helium, ∼60% of the A-sites become occupied. Helium uptake was quantified by both neutron powder diffraction and gas insertion and release measurements. After the conversion of gauge pressure to fugacity, the uptake of helium by CaNbF_6_ can be described by a Langmuir isotherm. The enthalpy of absorption for helium in [He_2–*x*_□_*x*_][CaNb]F_6_ is estimated to be ∼+3(1) kJ mol^–1^, implying that its formation is entropically favored. Helium is able to diffuse through the material on a time scale of minutes at temperatures down to ∼150 K but is trapped at 100 K and below. The insertion of helium into CaNbF_6_ reduces the magnitude of its negative thermal expansion, increases the bulk modulus, and modifies its phase behavior. On compressing pristine CaNbF_6_, at 50 and 100 K, a cubic (Fm3̅m) to rhombohedral (R3̅) phase transition was observed at <0.20 GPa. However, a helium-containing sample remained cubic at 0.4 GPa and 50 K. CaNbF_6_, compressed in helium at room temperature, remained cubic to >3.7 GPa, the limit of our X-ray diffraction measurements, in contrast to prior reports that upon compression in a nonpenetrating medium, a phase transition is detected at ∼0.4 GPa.

## 1 Introduction

The chemistry of helium in the solid state is limited in scope but of practical and fundamental interest.^1^ For example, α decay in nuclear fuel results in the formation of helium that can, in principle, lead to bubble formation, fuel element swelling, and possibly failure. Consequently, there have been studies of the solubility and energetics of helium incorporation in materials such as actinide dioxides, and also its diffusion through the materials.^2−8^ There has also been interest in the formation of helium compounds with iron, and iron oxide, in the context of exoplanet interiors and deep earth minerals,^9−11^ as these materials could be important in accounting for helium abundance in the earth and solar system. The boundaries of helium chemistry have been further expanded by preparing the electride Na_2_He at >113 GPa.^12^ There are computational reports of many other exotic helium baring phases, for example, CaF_2_He and Li_2_OHe,^13^ and compounds based on alkali metal sulfides.^14^

Helium is quite widely used as a pressure-transmitting medium in high-pressure experiments, as it has a low freezing point and sustains hydrostatic conditions to higher pressures than alternative media.^15^ However, helium is the prototypical penetrating medium as it readily diffuses under high pressures into a wide range of materials, altering their properties or even forming new compounds. For example, it can be incorporated into aluminosilicates, silica glasses,^16−20^ and crystalline silica.^21−23^ It can also be inserted into arsenolite to form a clathrate, As_4_O_6_·2He.^24,25^ Helium is also known to form clathrates with water.^26−29^ Notably the helium fullerene, He@C_60_, has attracted attention, in part, due to the quantum behavior seen in this “particle in a box” system.^30,31^

In 2017, we reported that helium readily inserted into the ReO_3_-type negative thermal expansion material CaZrF_6_ at room temperature and a mere 0.5 GPa and that the resulting defect double perovskites [He_2–*x*_□_*x*_][CaZr]F_6_ could be recovered to ambient pressure at low temperature.^32^ We also reported how the incorporation of helium changes the properties of the CaZrF_6_ framework.^33^ In a recent computational study, the incorporation of helium into a range of ReO_3_-type materials to form perovskites, including He_2_[CaZr]F_6_, and other phases has been examined, and the thermodynamics explored.^34^ The authors concluded that entropy can play an important role in stabilizing helium-containing perovskites.

In the current work, we employ neutron and X-ray powder diffraction to examine the incorporation of helium into CaNbF_6_ and subsequent defect perovskite formation, and how the incorporation of helium changes both the thermophysical properties of the material and its phase behavior. We also pay particular attention to quantifying helium uptake as a function of pressure, and provide an experimental estimate for the enthalpy change associated with helium insertion to form [He_2–*x*_□_*x*_][CaNb]F_6_.

## 2 Methods

### 2.1 Syntheses

NbF_4_ was prepared using a procedure based on that reported by Chassaing and Bizot.^35^ Nb metal powder (Alfa Aesar, 99.99%) and NbF_5_ (STREM, 99.5%) were mixed and ground in a 1:10 ratio by weight under a dry nitrogen atmosphere. A 4 g sample of the mixture was loaded into a nickel tube, which was sealed by arc welding under argon. The tube was heated at 300 °C for 4 days and then quenched in an iced water bath. Excess NbF_5_ was sublimed out of the product mixture under vacuum, using an oil bath temperature of 120 °C. The purity of the resulting NbF_4_, which was a dark black powder, was checked by powder diffraction. The material used to make the CaNbF_6_ sample for the neutron experiment was pure by diffraction, but contamination with small amounts of residual NbF_5_ can be a problem with this method.

CaNbF_6_ samples for the neutron diffraction and direct gas uptake measurements were prepared using a procedure based on that reported by Goubard et al.^36^ CaF_2_ (Alfa Aesar, 99.95%) and NbF_4_ were mixed and ground in a 1:1 molar ratio under a dry nitrogen atmosphere. A 1 g sample of the mixture was loaded into a nickel tube, which was then sealed by arc welding under argon. The nickel tube was then sealed inside an evacuated fused quartz ampule. The ampule was heated at 520 °C for 5 days and then cooled to room temperature over 24 h. The resulting product was a light gray powder.

CaNbF_6_ for the high-pressure X-ray diffraction measurement was prepared from NbF_5_ (99.5%, STREM), Nb (99.8%, Alfa Aesar), and CaF_2_ (99.5%, Sigma-Aldrich) via direct solid-state reaction using a procedure adapted from Chassaing et al.^37^ A 5:4:1 molar ratio of CaF_2_/NbF_5/_Nb was ground together under dry nitrogen and then placed into a nickel tube, which was sealed by arc welding under argon. The nickel tube was then sealed in an evacuated fused silica ampule. The ampule was heated from room temperature to 300 °C, held at 300 °C for 48 h, heated to 520 °C, held at 520 °C for 72 h, and cooled down from 520 °C to room temperature over 24 h. The product was a gray powder.

### 2.2 High-Pressure Neutron Diffraction

Measurements were performed at the Spallation Neutron Source, ORNL, using the instrument SNAP. The detectors were positioned at 50 and 90 deg and an evacuated flight tube, rather than a focusing guide, was employed. The sample was contained in an aluminum body gas pressure cell with a maximum working pressure of 450 MPa. The helium pressure was adjusted and monitored via a Harwood Engineering gas panel. A radial collimator with gadolinium blades was clamped on the pressure cell to reduce the scattering background from the cell’s body. The pressure cell was mounted on a sample stick and placed in a top-loading cryostat. Data were recorded over the pressure and temperature ranges 0–400 MPa and 50–295 K, respectively.

### 2.3 Gas Uptake and Release Measurements

The helium content of the [He_2–*x*_□_*x*_][CaNb]F_6_ samples, prepared under different helium pressures while cooling to 100 K from close to room temperature, was estimated by evacuating the head space over the sample at 100 K, sealing the head space, and then warming the pressure cell while monitoring the pressure in the head space. As the head space volume had previously been determined by gas expansion into a calibrated volume, and the mass of the sample was known, the resulting pressure after warming could be used to calculate the amount of helium released from the sample. These measurements were performed using an aluminum body high-pressure gas cell similar to that used for the neutron diffraction study. The cell, containing the sample, was mounted on a sample stick and placed in a top-loading cryostat. The sample was then cooled to 100 K under a constant pressure of helium gas at the maximum rate allowed by the cryostat. This cooldown took several hours. After evacuating the sample head space, it was sealed and the cell was warmed from 100 to 300 K in 25 K steps while recording the head space pressure.

### 2.4 High-Pressure X-ray Diffraction

High-pressure powder X-ray diffraction data were collected at the 17-BM beamline of the Advanced Photon Source, Argonne National Laboratory. The data were recorded on a PerkinElmer amorphous silicon 2D detector using a wavelength of 0.45428 Å. The measurements used an ∼100 μm diameter X-ray beam. The sample was compressed in a BX-90 diamond anvil cell equipped with 800 μm culet diamonds and a stainless-steel gasket (400 μm hole). Helium, loaded using the high-pressure gas loading system at GSECARS, was used as a pressure-transmitting medium.^38^ The known equation of state and the measured lattice constant of NaCl were used to determine the pressure.^39^

### 2.5 Diffraction Data Analysis

**Figure 1:**
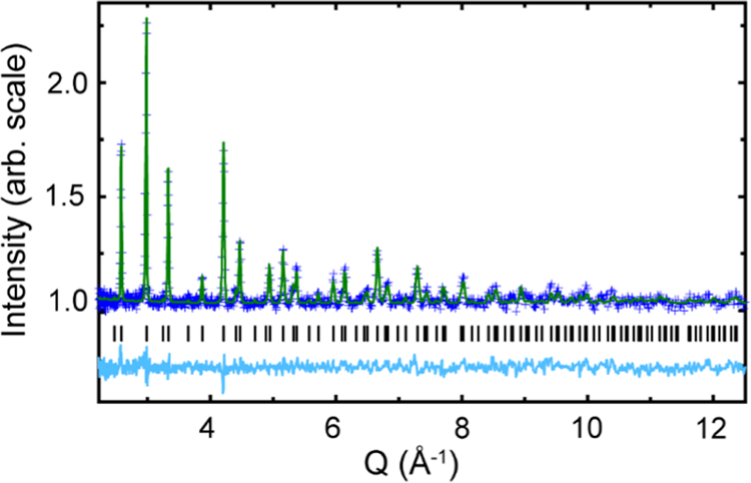
Rietveld fit to the neutron diffraction data for cubic [He_2–*x*_□_*x*_][CaNb]F_6_ acquired at 50 K and 0.30 GPa after cooling from 295 to 50 K in 0.30 GPa helium. The blue crosses are the data, the green line is the fit, the pale blue line is the difference curve, and the black “tick marks” indicate the expected Bragg peak positions.

The powder neutron diffraction data were analyzed using the Rietveld method in the GSAS-II (General Structure Analysis System-II) software package.^40^ The neutron data were of sufficient quality to facilitate a complete structure refinement including helium site occupancies. An example Rietveld fit to some neutron diffraction data is shown in Figure 1. A summary of key information from the fitting is provided in Table S1.

The 2D high-pressure X-ray powder diffraction data were integrated, and the resulting 1D data was background subtracted using DIOPTAS.^41^ Rietveld analyses were performed using GSAS, coupled with EXPGUI, to determine unit cell volume as a function of pressure.^42,43^ An example Rietveld fit is shown in Figure S1.

## 3 Results and Discussion

### 3.1 High-Pressure Neutron Diffraction

**Scheme 1:**
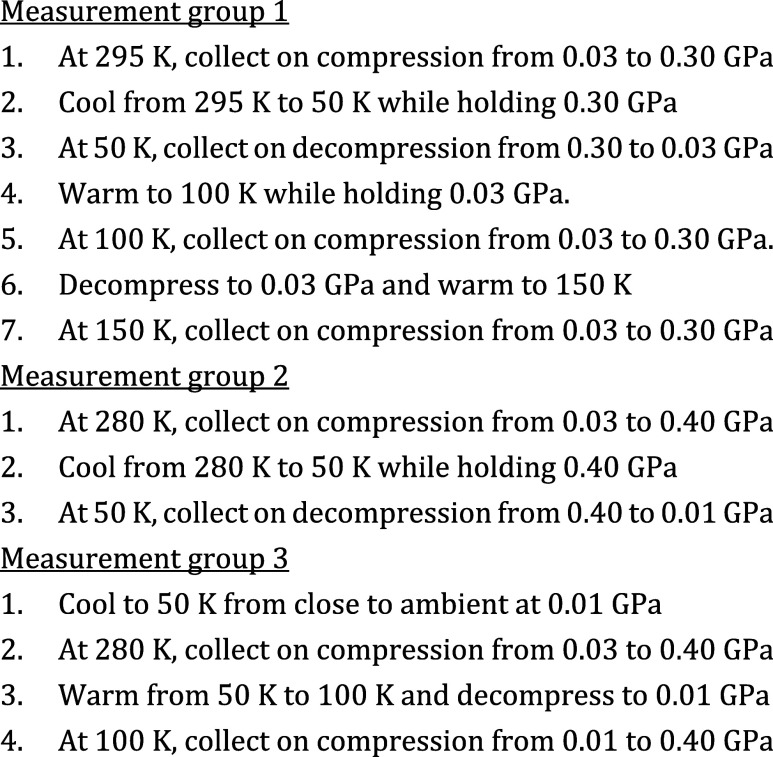
Path through Pressure–Temperature Space while Conducting the Neutron Powder Diffraction Measurements

Unit cell volumes were determined as a function of pressure and temperature from three groups of powder neutron diffraction measurements, on the same sample of CaNbF_6_, in a high-pressure helium atmosphere. The measurement sequence and conditions are laid out fully in Table S1 and summarized in the following Scheme 1.

**Figure 2:**
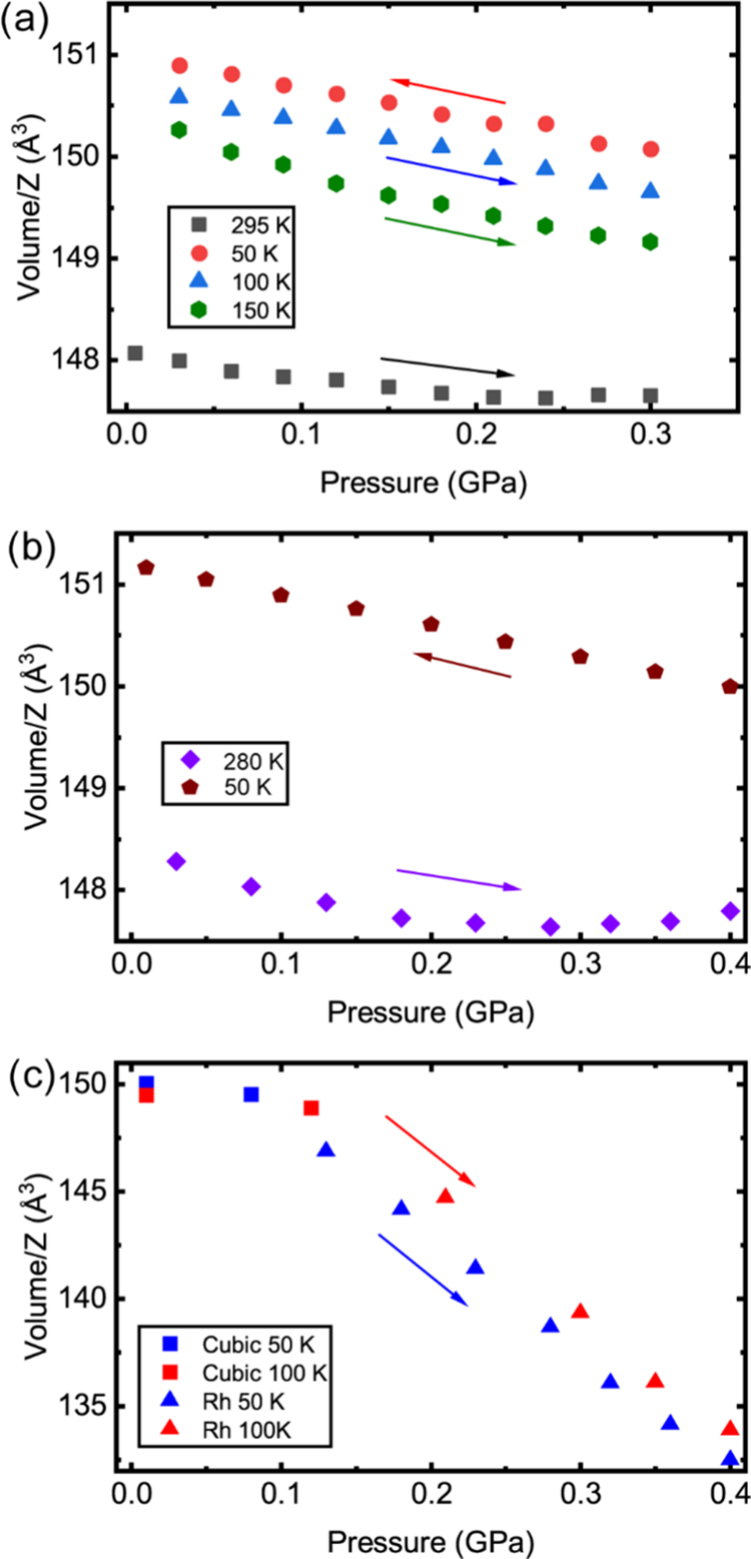
Unit cell volume per formula unit (V/Z) for [He_2–*x*_□_*x*_][CaNb]F_6_ as a function of helium pressure and temperature. The arrows in the panels indicate compression or decompression as the diffraction measurements were made. (a) Values resulting from measurement group 1, where the sample was cooled from 295 to 50 K while maintaining a helium pressure of 0.30 GPa (see Scheme 1). (b) Values resulting from measurement group 2, where the sample was cooled from 280 to 50 K while maintaining a helium pressure of 0.40 GPa (see Scheme 1). (c) Values resulting from measurement group 3, where the sample was cooled from close to ambient to 50 K, under 0.01 GPa helium pressure (see Scheme 1).

V/Z (unit cell volume per formula unit), as determined from the neutron powder diffraction measurements, is shown in Figure 2.

**Figure 3:**
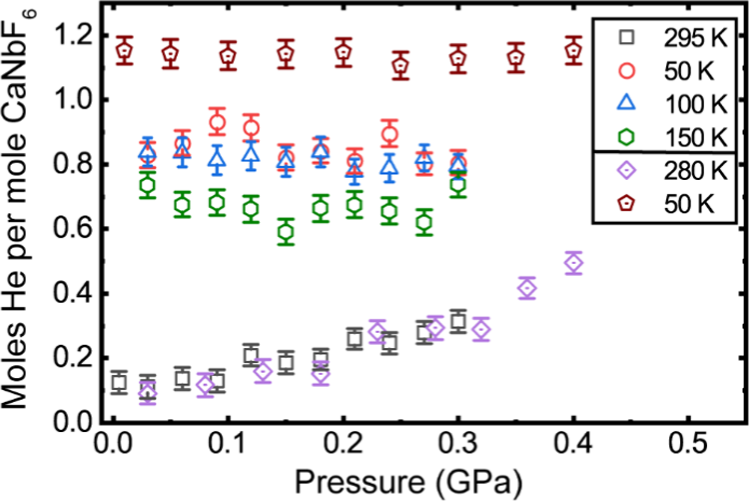
Helium content for [He_2–*x*_□_*x*_][CaNb]F_6_, as estimated from the Rietveld analyses of the neutron diffraction data using a site occupancy refinement. The order in which the temperatures are listed in the legend reflects the order of the measurements. The first group of measurements involved a cooldown from 295 to 50 K under 0.30 GPa helium (as laid out in Scheme 1) and the second group of measurements involved a cooldown from 280 to 50 K under 0.40 GPa helium (as laid out in Scheme 1). Note that as there are two moles of A-sites per mole of CaNbF_6_, complete filling of the A-sites with one helium (site occupancy of 1.0) corresponds to two moles of helium per mole of CaNbF_6_.

As previously reported for CaZrF_6_, V/Z does not vary linearly on compression in helium at close to room temperature (295 and 280 K in Figure 2a,b).^32,33^ In particular, upon compression at 280 K, it starts to increase above ∼0.28 GPa. This signals that the helium is inserting into the CaNbF_6_ to form [He_2–*x*_□_*x*_][CaNb]F_6_ and “inflating” the unit cell. At temperatures as low as 150 K, the variation of V/Z with pressure is still nonlinear (Figure 2a), indicating that helium can still enter/exit the structure. Upon pressure change at and below 100 K (Figure 2a,b), the unit cell volume varies linearly with pressure suggesting that the helium is trapped within the structure on the time scale of the neutron diffraction measurements. As helium has a significant neutron scattering length (*b*_He_ = 3.26 fm, *b*_Ca_ = 4.70 fm, *b*_Nb_ = 7.05 fm, *b*_F_ = 5.65),^44^ the ordered arrangement of helium in the material affects the peak intensities in the neutron powder diffraction pattern, enabling helium site occupancy quantification from the diffraction data (see Figure 3).

The helium content of the material, [He_2–*x*_□_*x*_][CaNb]F_6_, was quantified by refining the occupancy of helium on the perovskite A-site with the atomic displacement parameter (ADP) for the helium constrained to be the same as the equivalent isotropic ADP for the fluoride, to reduce the correlation between the site occupancy and its ADP. Other constraint schemes for the helium ADP were explored. For example, constraining it to be equivalent to the ADP of the calcium, rather than fluorine, gave helium occupancies that were different by a few %, but the one adopted gave occupancies in excellent agreement with the helium content measured directly by gas uptake and release (see Section 3.2). Results from the site occupancy refinement are shown in Figure 3. On compressing the sample in helium at close to room temperature (280 and 295 K), the refined site occupancy climbed smoothly, but not linearly. The initial site occupancy at zero pressure of close to 0.06 (Table S1) is due to systematic error in the analyses. On cooling under pressure to 50 K, the site occupancies increase significantly, which is likely due to an increase in fugacity on cooling at constant gauge pressure (see later). Cooling under 0.4 GPa helium gave a site occupancy of just under 60% and cooling under 0.3 GPa led to an occupancy of just above 40%. These occupancies are in very good agreement with those determined independently from gas uptake and release measurements—see Section 3.2. On decompression at 50 K, after cooling under 0.4 GPa helium, the refined occupancies are essentially constant, indicating that on the time scale of the neutron diffraction measurement, the helium is trapped in the material. Similarly, on decompression at 50 K, after cooling under 0.3 GPa helium, the refined occupancies show no systematic variation with pressure. Measurements after warming up from 50 to 100 K (blue triangles in Figure 3) show little variation of site occupancy with pressure, suggesting that the helium remains trapped, for kinetic reasons, inside the structure as the pressure is changed. However, after warming to 150 K, the site occupancy is not constant on compression (green circles in Figure 3), and it is lower than at both 50 and 100 K, indicating that at this temperature, there is slow movement of helium in and out of the structure on the time scale of the measurement. This interpretation is supported by our direct gas uptake and release measurements—see Section 3.2.

**Figure 4:**
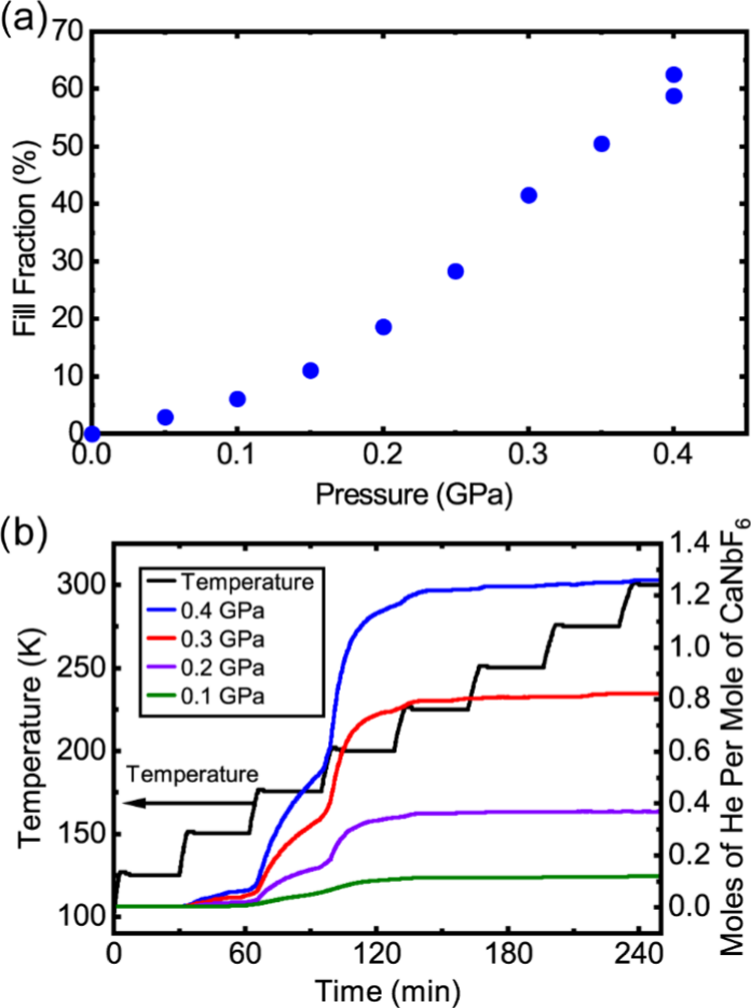
(a) Amount of helium released on heating to 300 K [He_2–*x*_□_*x*_][CaNb]F_6_, which had been prepared by cooling from ∼300 to 100 K under different applied helium pressures. (b) Amount of helium released, as a function of time/temperature, for [He_2–*x*_□_*x*_][CaNb]F_6_ as the samples were warmed to room temperature in 25 K steps.

The data shown in Figure 2a indicate that the material obtained by cooling under 0.3 GPa helium shows significant negative thermal expansion (NTE). From the measurements at 50 and 100 K, the extrapolated zero pressure volume coefficient of thermal expansion (CTE) at 75 K is estimated to be −40(3) × 10^–6^ K^–1^ (see Figure S2), which is significantly less in magnitude than that reported for helium-free CaNbF_6_ at 75 K (−65 × 10^–6^ K^–1^).^45^ This reduction in NTE magnitude on inserting helium is consistent with our prior observations for CaZrF_6_.^32,33^ Likely, the presence of helium in the A-site (40–45% fractional occupancy for this sample—see Figures 3 and 4) sterically impedes the transverse vibrational motion of the fluoride, which is what underlies NTE in materials of this type,^46^ reducing the magnitude of the NTE, but not eliminating it. It is unclear if 100% helium occupancy would eliminate the NTE. Our estimate for the pressure dependence of the volume CTE (−50(15) × 10^–6^ K^–1^/GPa, see Figure S2) suggests that compression of the material increases the magnitude of the NTE. This is consistent with prior observations for Zn(CN)_2_^47^ and has also been reported for CaZrF_6_.^48^ Compression likely softens the potential for transverse motion of the fluoride, leading to the NTE enhancement.

The insertion of helium into CaNbF_6_ also affects bulk moduli. The bulk modulus of the sample cooled under 0.4 GPa helium is estimated to be 49.7(4) GPa at 50 K, which is slightly stiffer than that of the sample cooled under 0.3 GPa helium at 50 K: 47.9(7) GPa. For reference, the bulk modulus of CaNbF_6_ was reported to be 33.7(4) GPa at room temperature^45^ and the current data suggest a bulk modulus of 20 GPa at 50 K, when the material is cooled so that no helium is incorporated. The much higher bulk modulus, at a low temperature (50 K), for the materials containing helium likely reflects a steric interaction between the helium and the framework, which stiffens the structure and suppresses the cubic to rhombohedral phase transition seen on compressing the sample containing no helium. The helium-containing material softens on heating as the bulk modulus at 100 K for the sample cooled under 0.3 GPa helium is estimated to be 43.9(7) GPa. Softening on heating is typical for most materials; CaZrF_6_, with no helium incorporated, has been reported to soften on heating by −0.012(2) GPa K^–1^ over the temperature range ca. 300–500 K.^48^

It is notable that the samples cooled under high-pressure helium (0.3 and 0.4 GPa) remained cubic down to the lowest temperatures studied, even though a phase transition has been reported on compressing CaNbF_6_^45^ in silicone oil to ∼0.4 GPa at room temperature. However, compression of CaNbF_6_ cooled to 50 K under 100 bar (0.01 GPa) helium led to a phase transformation from cubic (Fm3̅m) to rhombohedral (R3̅). Apparently, the insertion of helium into the structure, by compression prior to cooling, suppresses phase transformation. A cubic to rhombohedral transformation is common on compressing or cooling cubic ReO_3_-type and double ReO_3_-type fluorides. For example, at 300 K, compression of ScF_3_ gives rise to a Pm3̅m to R3̅c transition at ∼0.7 GPa,^49,50^ compression of MgZrF_6_ at 300 K leads to a Fm3̅m to R3̅ transition at ∼0.35 GPa,^45^ while cooling at ambient pressure leads to the same transition at ∼100 K,^45^ and compression of NaNbF_6_ at 300 K leads to a Fm3̅m to R3̅ transition at ∼0.3 GPa while cooling at ambient pressure leads to the same transition at ∼130 K.^51^ These cubic to rhombohedral transitions involve tilting of the octahedra that make up the ReO_3_ framework (Glazer type a^–^a^–^a^–^)^52^ and no change in bonding. However, compression of CaNbF_6_ in silicone oil at room temperature has previously been reported to lead to an as-yet-unidentified crystal structure.^45^ This transformation likely involves a change in bonding for one or more metal ions, perhaps similar to that reported on going from cubic to tetragonal CaZrF_6_,^53^ rather than the octahedral tilting transformation commonly seen in other materials, and also seen in the current study when the sample was compressed at both 50 and 100 K with no inserted helium. The difference in behavior on compression at low temperature, and compression at room temperature, may be due to kinetics. Making and breaking bonds during a reconstructive phase transformation of the type reported to occur at 300 K in the absence of helium may require thermal energy to overcome an activation barrier, which is not readily available at low temperature.

### 3.2 Gas Uptake and Release

Helium uptake and release by CaNbF_6_ were examined at several pressures up to 0.4 GPa. These measurements allow the direct quantification of helium in the structure after cooling under pressure followed by pressure release (Figure 4a) and also provide a qualitative picture of the kinetics for helium migration out of the material (Figure 4b).

Examination of Figure 4b shows that, on the time scale of the measurement, helium is effectively trapped inside the [He_2–*x*_□_*x*_][CaNb]F_6_ for temperatures of 125 K and lower, but, at 150 K, helium is slowly released from the samples. This is fully consistent with the results from our neutron diffraction measurements shown in Figures 2 and 3. At 200 K, the release of gas is quite rapid. Surprisingly, helium release from the sample seems to occur at a slightly lower temperature than was observed for CaZrF_6_ (onset at ∼175 K), even though CaZrF_6_ has a larger unit cell than CaNbF_6_.^32^ This may be due to differences between the surface of the CaZrF_6_ and CaNbF_6_ grains; CaZrF_6_ is known to amorphize quite readily on grinding,^33,48,53^ so its grains may have a disordered surface layer, which could impede gas transport in and out of the grain interior. When considering the pore sizes in these materials, it is notable that twice the van der Waals radius of helium (2 × 1.40 = 2.80 Å) is much larger than the open pore size, if that is estimated as the fluorine-to-fluorine distance minus twice the van der Waals radius of fluorine. For CaNbF_6_, the later distance would be approximately [4.21 – (2 × 1.47)] = 1.27 Å.

The amount of helium trapped in [He_2–*x*_□_*x*_][CaNb]F_6_ on cooling from room temperature to 100 K, shown in Figure 4a, is not a true equilibrium measurement for a well-defined temperature, as the sample cooldown was quite slow due to the high heat capacity of the sample cell and the limited cooling power of the cryostat. On cooling in high-pressure helium, the solid sample will continue to exchange helium with the high-pressure gas until the kinetics become prohibitively slow. Based on the data shown in Figure 4b, the solid sample will likely fall out of equilibrium with the gas between 175 and 125 K during cooldown. As the cooldown process was similar for each set of measurements, the effective “equilibrium” temperature for each point in Figure 4a should be comparable.

**Figure 5:**
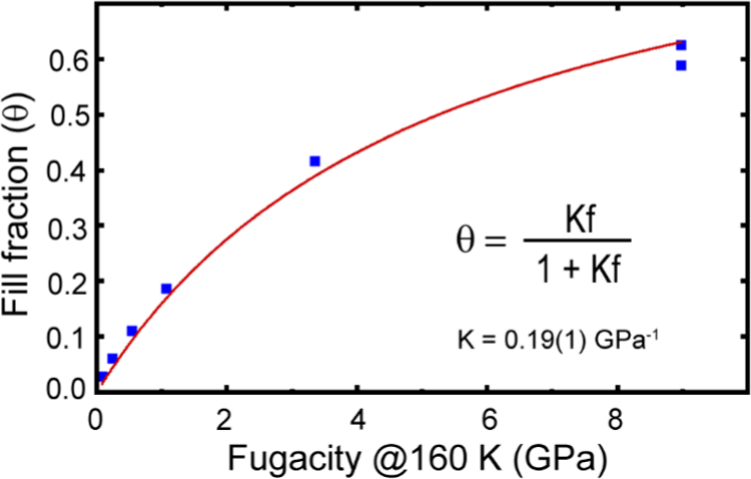
Langmuir isotherm fit to fill fraction versus fugacity for [He_2–*x*_□_*x*_][CaNb]F_6_. The fill fractions are from the gas uptake and release measurements, and the fugacities were calculated from the gauge pressures, assuming an effective equilibration temperature of 160 K.

The shape of the curve shown in Figure 4a is, at first sight, surprising. The amount of gas trapped in clathrate-type materials, as a function of gas loading pressure, is often well described by a Langmuir isotherm, where the fill fraction of the available sites in the clathrate increases rapidly at low pressures and then asymptotically approaches unity at higher pressures. See, for example, work examining molecular-hydrogen storage in THF–H_2_ clathrate hydrates by Strobel et al.^54^ The difference in shape between the curve shown in Figure 4a and a Langmuir isotherm is likely a consequence of the nonideality of helium under the low-temperature and high-pressure conditions used to prepare [He_2–*x*_□_*x*_][CaNb]F_6_. Using thermodynamic data for helium at low temperatures and high pressures as reported in NIST Technical Note 1334 (revised),^55^ fugacities for helium in the temperature and pressure range of the performed measurements were calculated. See the Supporting Information for further details. At the temperatures and pressures relevant to the gas loading measurements, the fugacities are dramatically higher than the gauge pressures (see Figure S4). In Figure 5, the data from Figure 4a are plotted versus fugacity rather than pressure, and a Langmuir isotherm fit to the values. A temperature of 160 K was assumed during the calculation of the fugacities for this plot, based on the kinetics for gas uptake and release shown in Figure 4b. The fit quality is quite good, suggesting that the shape of Figure 4a is largely due to the nonideal behavior of helium rather than any novel physical process.

Isotherms recorded at different temperatures can be used to estimate enthalpies of adsorption.^56,57^ While the gas uptake and release measurements only provide fill fraction versus fugacity for one temperature, the neutron-derived site occupancies shown in Figure 3 provide additional data for higher temperatures. A Langmuir isotherm was fit to site occupancies, from the 280 K neutron diffraction measurements, versus fugacity (Figure S7). This gave a best fit estimate for the equilibrium constant at 280 K, K_280 K_, of 0.48(3) GPa^–1^. For the latter isotherm, the fugacities were estimated by interpolation of the thermodynamic data (Figure S6) in Arp et al., NIST Technical Note 1334 (revised).^55^ Assuming that the enthalpy of adsorption for helium in [He_2–*x*_□_*x*_][CaNb]F_6_ is independent of fill fraction and temperature, and K_280 K_ and K_160 K_ can be treated as thermodynamic equilibrium constants, application of the van’t Hoff equation gives an enthalpy estimate for the uptake of helium by CaNbF_6_ of +3(1) kJ mol^–1^. The biggest contributor to the uncertainty in this enthalpy estimate is the uncertainty in the effective equilibrium temperature, which was taken as ±15 K, associated with the gas trapping measurements. This suggests that the formation of [He_2–*x*_□_*x*_][CaNb]F_6_ from high-pressure helium and CaNbF_6_ is driven entropically rather than enthalpically. In a computational study of [He_2_][CaZr]F_6_, and various HeMF_3_ (M = trivalent metal) perovskites, the authors concluded that entropy can stabilize the formation of such perovskites because the available volume within the perovskite pores may be greater than the volume per atom in high-pressure elemental helium. However, their calculations, including dispersion interactions, suggested a slightly negative enthalpy of formation for [He_2_][CaZr]F_6_ from helium and CaZrF_6_ (−11 meV/atom, or −1 kJ mol^–1^, at zero pressure).^34^ For comparison, the experimental isosteric heat of adsorption for helium on graphite at 18.5 K and 20% coverage has been reported as −1.7 kJ mol^–1^.^58^

### 3.3 High-Pressure Powder X-day Diffraction

**Figure 6:**
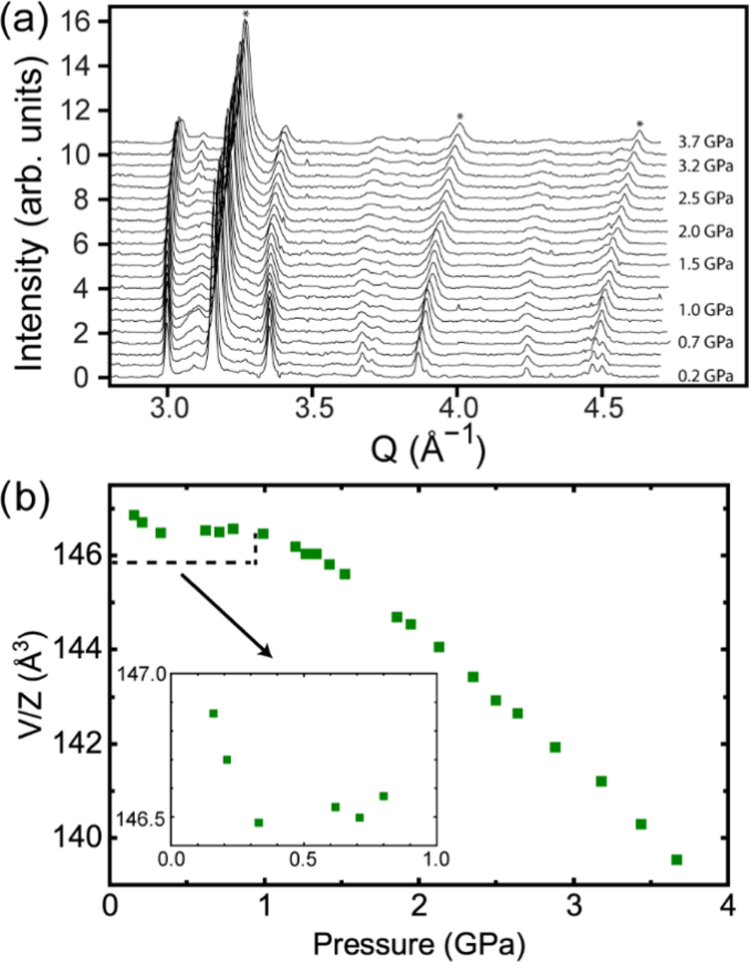
(a) High-pressure X-ray diffraction data. The peaks from the NaCl pressure standard are marked *. (b) Unit cell volume per formula unit versus pressure for CaNbF_6_ compressed in helium.

The material in this section, and the corresponding components of the experimental section, have previously been reported in the PhD thesis of Dr. Brett Hester.^59^ X-ray measurements using a diamond anvil cell allowed access to much higher pressures than were possible with the gas cell for neutron diffraction. The high-pressure diffraction data for CaNbF_6_ in helium (Figure 6a) indicate that the gas is inserted into the structure as the pressure is increased, in a similar fashion to CaZrF_6_.^33^ At low pressure, the unit cell volume initially decreases and then increases before going through a maximum at ∼0.9 GPa (Figure 6b). This unusual behavior confirms the insertion of helium into the ReO_3_-type structure of CaNbF_6_, which creates a perovskite material with helium on the A-site. It is possible that above ∼1.0 GPa, the material is close to stoichiometric with helium giving rise to [He_2_][CaNb]F_6_, but owing to the small X-ray scattering cross section of helium, this cannot be verified. A linear fit to the natural logarithm of volume, ln(*V*), versus *P* in the pressure range from 1.4 to 3.7 GPa suggests that the perovskite has a bulk modulus of 51.47(2) GPa, which is slightly higher than that found for [He_2_][CaZr]F_6_ (∼47 GPa)^33^ over a similar pressure range.

In nonpenetrating media, on compression at room temperature, CaNbF_6_ has been reported to undergo a crystalline to crystalline phase transition at ∼0.4 GPa and amorphization at ∼4 GPa.^45^ In the current experiment, the perovskite resulting from helium insertion is stable up to the highest pressure recorded of ∼3.7 GPa. However, there is some reduction of peak intensity on compression, which might indicate an onset of amorphization at pressures just above 3.7 GPa.

The diffraction data for CaNbF_6_ show some quite broad peaks at low Q (∼1.20, 1.55, and 1.85 Å^–1^) from an impurity phase (Figure S1). They may arise from the creation of a new high-pressure phase when grinding or pressurizing the sample. This type of impurity has been previously reported for ZnNbF_6_ and was also seen to a lesser extent for CaZrF_6_.^33,60^ Their presence did not impede the analysis of the data for the main phase.

## 4 Conclusions

Cooling CaNbF_6_ in the presence of high-pressure helium results in the formation of the defect perovskite [He_2–*x*_□_*x*_][CaNb]F_6_. This new phase shows different thermal expansion, bulk modulus, and phase behavior when compared with the pristine material, presumably due to steric interactions between helium residing on the dodecahedral A-site and the fluoride, which modifies the lattice dynamics underpinning both the negative thermal expansion and the cubic to rhombohedral phase transition seen on compression of the parent CaNbF_6_ at low temperatures. Helium uptake, to form [He_2–*x*_□_*x*_][CaNb]F_6_, can be described by a Langmuir isotherm, but only after the gauge pressure is converted to fugacity, as helium is highly nonideal in this pressure/temperature regime leading to the fugacity being much higher than the gauge pressure. Our estimate of the enthalpy for helium uptake to form [He_2–*x*_□_*x*_][CaNb]F_6_, +3(1) kJ mol^–1^, suggests that its formation is entropically favored, which is in agreement with prior predictions.^34^

## Supporting Information Available

Additional Rietveld plots, pressure dependence of CTE, fits to determine bulk moduli, estimates of fugacity versus pressure for different temperatures, A-site fill fraction from gas uptake and release plotted versus fugacity assuming a temperature of 180 K, fits to thermodynamic data for helium, Langmuir isotherm fit, unit cell dimensions and A-site occupancies derived from the neutron diffraction data, tabulated bulk moduli, and unit cell dimensions for CaNbF_6_ from the high-pressure X-ray measurements (PDF)
