# Supplementary material for: Synthesis and Properties of the Helium Clathrate and Defect Perovskite [He2–x□x][CaNb]F6
Source: J Phys Chem C Nanomater Interfaces. 2024 Jun 21;128(26):11006–13. doi: 10.1021/acs.jpcc.4c02174 (PMC11229063; doi:10.1021/acs.jpcc.4c02174)
Supplement: Supplementary file 1 — jp4c02174_si_001.pdf [file jp4c02174_si_001.pdf]

Supplementary material for "Synthesis and Properties of the Helium Clathrate and Perovskite $[He_{2-x}\square_x][CaNb]F_6$"

Shangye Ma,[†] Brett R. Hester,[†] Anthony J. Lloyd II,[†] Antonio M. dos Santos, [‡] Jamie J. Molaison[‡] and Angus P. Wilkinson*[,†,§]

[†] School of Chemistry and Biochemistry, Georgia Institute of Technology, Atlanta, GA 30332-0400, United States

[‡]Neutron Scattering Division, Oak Ridge National Laboratory, Oak Ridge, TN 37831, United States

[§]School of Materials Science and Engineering, Georgia Institute of Technology, Atlanta, GA 30332-0245, United States

Contents:

Additional descriptive text

Estimation of fugacities.

List of Supplementary Figures

Figure S1. Rietveld plot showing a fit of the $CaNbF_6$ $Fm\bar{3}m$ model to the powder X-ray diffraction data obtained in a diamond anvil cell at an initial helium gas loading pressure of 0.16 GPa. The high-Q section has been scaled to show detail but shifted such that the background levels match. The phase tick marks denote $CaNbF_6$ (magenta) and NaCl (black).

Figure S2. Linear fit to the volumetric CTE for ~75 K versus pressure for $[He_{2-x}\square_x][CaNb]F_6$ prepared by cooling under 0.3 GPa helium. The CTE values were estimated from the difference between the 100 and 50 K unit cell volumes at the different pressures used in the diffraction measurements. The extrapolated volume CTE at zero pressure is estimated to be $-40(3) \times 10^{-6}$ K^{-1} and the slope $-50(15) \times 10^{-6}$ K^{-1} GPa^{-1}.

Figure S3. Linear fits to V versus P used to determine the bulk moduli in Table S2. a) Unit cell volume versus P for a sample loaded with helium at 0.3 GPa and measured at 50 K, b) unit cell volume versus P for a sample loaded with helium at 0.3 GPa and measured at 100 K, and c) unit cell volume versus P for a sample loaded with helium at 0.4 GPa and measured at 50 K.

Figure S4. Fugacity versus pressure at different temperatures, estimated from the thermodynamic data reported by Arp et al.[1]

Figure S5. Measured fill fraction for $[He_{2-x}\square_x][CaNb]F_6$, from the reported gas uptake and release measurements, versus the calculated helium fugacity using a temperature of 180 K when converting applied pressure to fugacity. A temperature of 160 K was used for the data shown in the main paper.

Figure S6. Fits to thermodynamic data, a) enthalpy and b) entropy, from Arp et al.[1] which were used to interpolate the data so that fugacities could be estimated for the high pressure neutron diffraction measurements at 280 K.

Figure S7. Langmuir isotherm fit to the 280 K neutron site occupancies as a function of fugacity.

List of Supplementary Tables

Table S1. Unit cell dimensions and A-site helium occupancy from the Rietveld fits to the powder neutron diffraction data.

Table S2. Bulk moduli for $[He_{2-x}\square_{x}][CaNb]F_6$ estimated from linear fits to V versus P.

Table S3. Unit cell volumes for cubic $CaNbF_6$ and NaCl as determined from Rietveld analyses of the high-pressure diffraction data for $CaNbF_6$ in a diamond anvil cell with a helium pressure medium. Pressures estimated from the unit cell volume of the NaCl using an equation of state are also given.[2]

Additional descriptive text

Estimation of fugacities

Free energies were calculated for helium either directly from the reported enthalpies and entropies in Arp et al.[1] or by interpolation of these data (see Fig. S6). These free energies were then used to estimate fugacities for helium at high pressure, assuming that at low pressure (0.01 MPa) fugacity and gauge pressure are equivalent.

Supplementary Figures

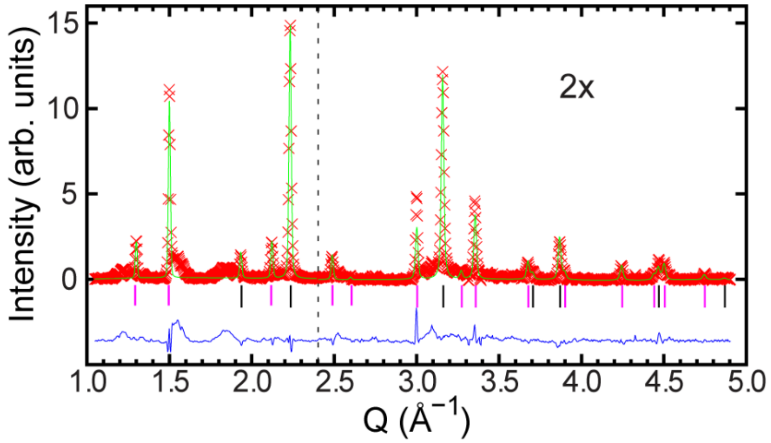

Figure S1. Rietveld plot showing a fit of the $CaNbF_6$ $Fm\overline{3}m$ model to the powder X-ray diffraction data obtained in a diamond anvil cell at an initial helium gas loading pressure of 0.16 GPa. The high-Q section has been scaled to show detail but shifted such that the background levels match. The phase tick marks denote $CaNbF_6$ (magenta) and NaCl (black).

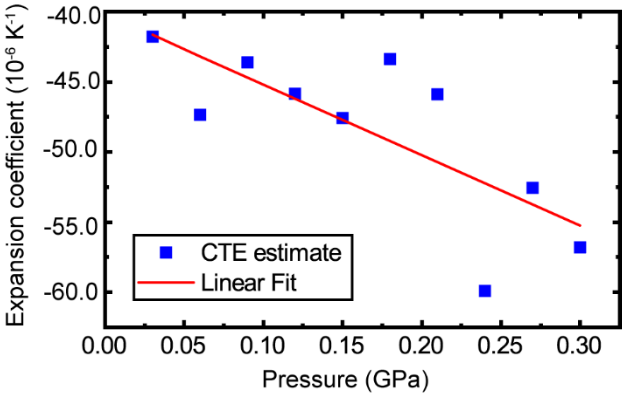

Figure S2. Linear fit to the volumetric CTE for ~75 K versus pressure for $[He_{2-x}\square_{x]}[CaNb]F_6$ prepared by cooling under 0.3 GPa helium. The CTE values were estimated from the difference between the 100 and 50 K unit cell volumes at the different pressures used in the diffraction measurements. The extrapolated volume CTE at zero pressure is estimated to be –40(3) × 10^{-6} K^{-1} and the slope –50(15) × 10^{-6} K^{-1} GPa^{-1}.

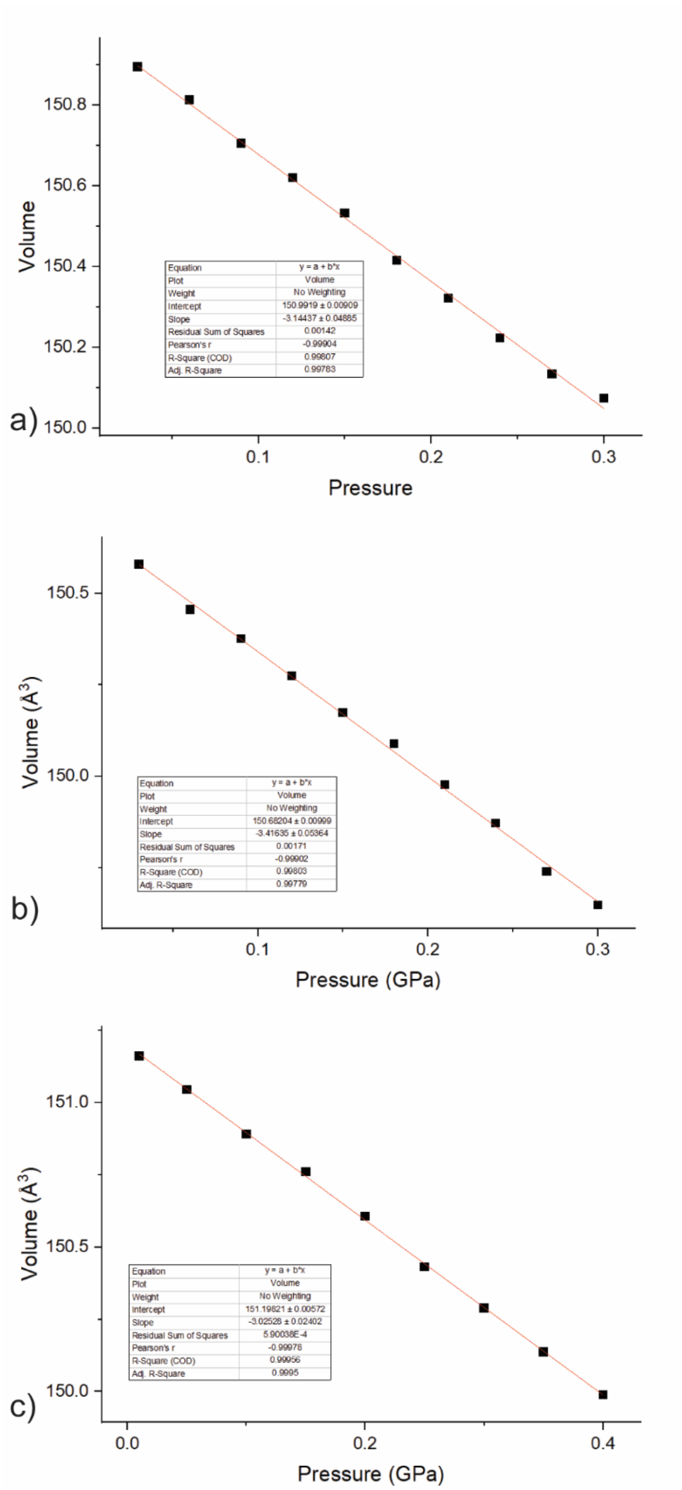

a)

Figure S3. Linear fits to V versus P used to determine the bulk moduli in Table S2. a) Unit cell volume versus P for a sample loaded with helium at 0.3 GPa and measured at 50 K, b) unit cell volume versus P for a sample loaded with helium at 0.3 GPa and measured at 100 K, and c) unit cell volume versus P for a sample loaded with helium at 0.4 GPa and measured at 50 K.

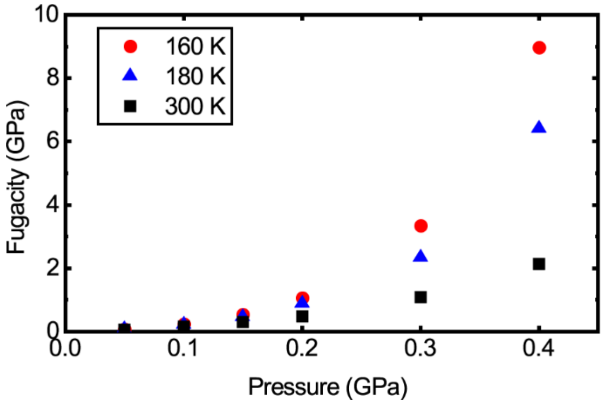

Figure S4. Fugacity versus pressure at different temperatures, estimated from the thermodynamic data reported by Arp et al.[1]

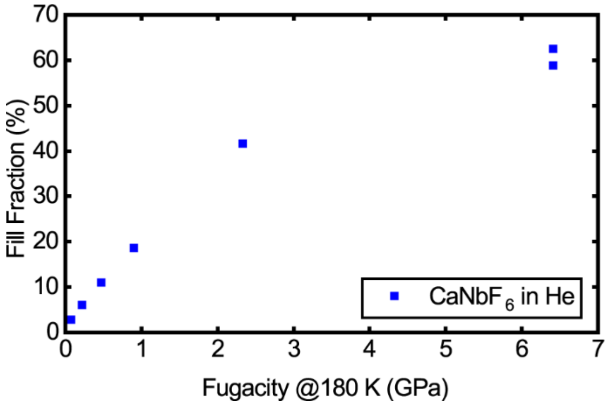

Figure S5. Measured fill fraction for $[He_{2-x}\square_{x}][CaNb]F_6$, from the reported gas uptake and release measurements, versus the calculated helium fugacity using a temperature of 180 K when converting applied pressure to fugacity. A temperature of 160 K was used for the data shown in the main paper.

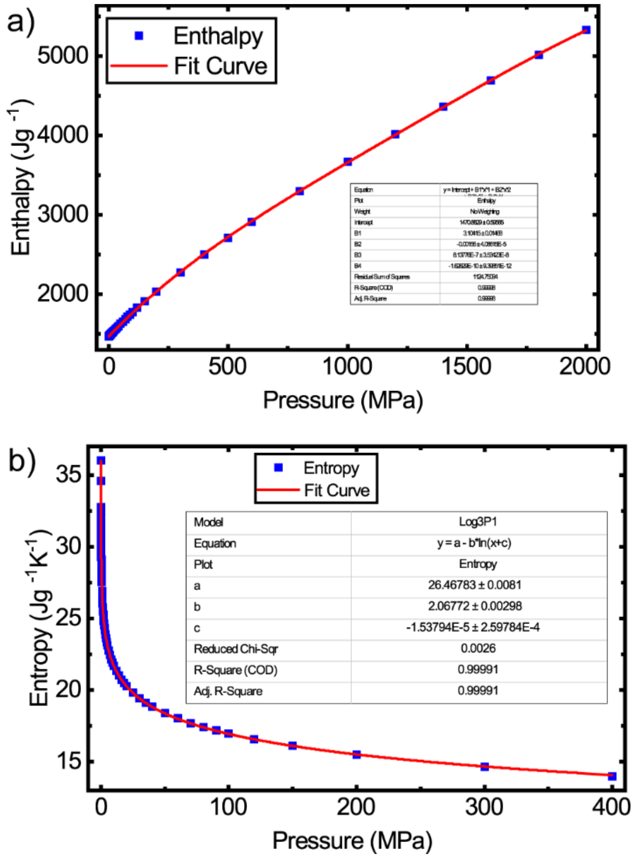

Figure S6. Fits to thermodynamic data, a) enthalpy and b) entropy, from Arp et al.[1] which were used to interpolate the data so that fugacities could be estimated for the high pressure neutron diffraction measurements at 280 K.

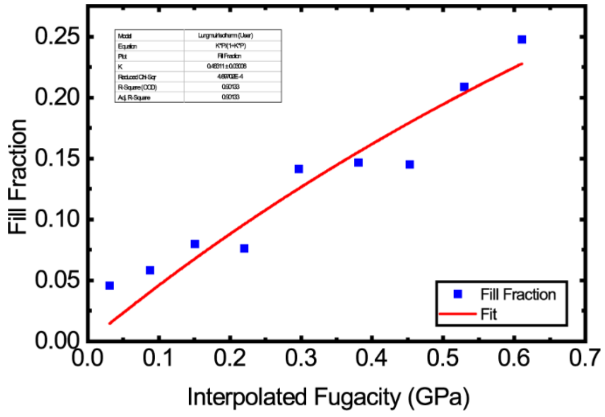

Figure S7. Langmuir isotherm fit to the 280 K neutron site occupancies as a function of fugacity.

Supplementary Tables

Table S1. Unit cell dimensions and A-site helium occupancy from the Rietveld fits to the powder neutron diffraction data.

**Conditions**	**Meas. #**	**T (K)**	**P (GPa)**	**a (Å)**	**c (Å) for rhombohedral phase**	**A-site fractional occupancy**
Compressed to 0.3 GPa at 295 K and then cooled to 50 K at 0.3 GPa	1	295	0.005	8.3980(2)		0.06(2)
	2	295	0.03	8.3966(2)		0.06(2)
	3	295	0.06	8.3947(2)		0.07(2)
	4	295	0.09	8.3936(2)		0.07(2)
	5	295	0.12	8.3931(2)		0.10(2)
	6	295	0.15	8.3918(2)		0.09(2)
	7	295	0.18	8.3906(2)		0.10(2)
	8	295	0.21	8.3898(2)		0.13(2)
	9	295	0.24	8.3896(2)		0.12(2)
	10	295	0.27	8.3902(2)		0.14(2)
	11	295	0.30	8.3902(2)		0.16(2)
	12	50	0.30	8.4357(3)		0.40(2)
	13	50	0.27	8.4368(3)		0.40(2)
	14	50	0.24	8.4385(3)		0.45(3)
	15	50	0.21	8.4404(3)		0.40(3)
	16	50	0.18	8.4421(3)		0.46(2)
	17	50	0.15	8.4443(3)		0.41(2)
	18	50	0.12	8.4459(3)		0.47(3)
	19	50	0.09	8.4475(3)		0.47(3)
	20	50	0.06	8.4495(2)		0.42(2)
	21	50	0.03	8.4511(3)		0.41(2)
	22	100	0.03	8.4452(2)		0.42(2)
	23	100	0.06	8.4429(2)		0.42(2)
	24	100	0.09	8.4414(3)		0.41(2)
	25	100	0.12	8.4395(2)		0.41(2)
	26	100	0.15	8.4376(2)		0.40(2)
	27	100	0.18	8.4360(3)		0.42(2)
	28	100	0.21	8.4339(2)		0.39(2)
	29	100	0.24	8.4319(2)		0.39(2)
	30	100	0.27	8.4294(2)		0.41(2)
	31	100	0.30	8.4277(2)		0.40(2)
	32	150	0.03	8.4392(2)		0.37(2)
	33	150	0.06	8.4352(2)		0.34(2)
	34	150	0.09	8.4328(2)		0.34(2)
	35	150	0.12	8.4293(2)		0.33(2)
	36	150	0.15	8.4272(2)		0.30(2)
	37	150	0.18	8.4255(2)		0.33(2)
	38	150	0.21	8.4235(2)		0.34(2)
	39	150	0.24	8.4216(2)		0.33(2)

	40	150	0.27	8.4197(2)		0.31(2)
	41	150	0.30	8.4186(2)		0.37(2)
Compressed to 0.4 GPa at 280 K and then cooled to 50 K at 0.4 GPa	42	280	0.03	8.4019(2)		0.05(2)
	43	280	0.08	8.3973(2)		0.06(2)
	44	280	0.13	8.3943(2)		0.08(2)
	45	280	0.18	8.3915(2)		0.08(2)
	46	280	0.23	8.3906(2)		0.14(2)
	47	280	0.28	8.3899(2)		0.15(2)
	48	280	0.32	8.3904(2)		0.14(2)
	49	280	0.36	8.3909(2)		0.21(2)
	50	280	0.40	8.3928(2)		0.25(2)
	51	50	0.40	8.4341(2)		0.58(2)
	52	50	0.35	8.4369(3)		0.57(2)
	53	50	0.30	8.4397(3)		0.56(2)
	54	50	0.25	8.4424(2)		0.55(2)
	55	50	0.20	8.4457(2)		0.57(2)
	56	50	0.15	8.4486(3)		0.57(2)
	57	50	0.10	8.4510(3)		0.57(2)
	58	50	0.05	8.4539(3)		0.57(2)
	59	50	0.01	8.4560(3)		0.58(2)
Cooled from high temperature to 50 K with 100 bar helium. Then compressed cold	60	50	0.03	8.435(3)		
	61	50	0.08	8.425(3)		
	62	50	0.13	5.896(5)	14.64(1)	
	63	50	0.18	5.831(5)	14.69(1)	
	64	50	0.23	5.762(9)	14.75(2)	
	65	50	0.28	5.69(1)	14.84(2)	
	66	50	0.32	5.62(2)	14.90(2)	
	67	50	0.36	5.58(2)	14.95(2)	
	68	50	0.40	5.53(2)	15.00(3)	
Warmed from 50 K to 100 K and decompressed. Then compressed cold	69	100	0.01	8.425(2)		
	70	100	0.12	8.414(3)		
	71	100	0.21	5.854(5)	14.63(1)	
	72	100	0.30	5.72(1)	14.76(2)	
	73	100	0.40	5.58(2)	14.91(3)	
	74	100	0.35	5.63(2)	14.86(2)	

Table S2. Bulk moduli for $[He_{2-x}\square_{x]}[CaNb]F_6$ estimated from linear fits to V versus P.

Sample conditions: Measurement temperature and helium pressure during cooling from near room temperature	Bulk Modulus (GPa)
50 K, 0.3 GPa	47.9(7)
100 K, 0.3 GPa	43.9(7)
50 K, 0.4 GPa	49.7(4)

Table S3. Unit cell volumes for cubic $CaNbF_6$ and NaCl as determined from Rietveld analyses of the high-pressure diffraction data for $CaNbF_6$ in a diamond anvil cell with a helium pressure medium. Pressures estimated from the unit cell volume of the NaCl using an equation of state are also given.[2]

$CaNbF_6$ a (Å)	$CaNbF_6$ Volume (Å^3)	NaCl Volume (Å^3)	Pressure (GPa)
8.3751(8)	587.44(17)	178.23(4)	0.16(8)
8.3720(8)	586.80(17)	177.86(4)	0.21(8)
8.3678(9)	585.92(19)	177.05(4)	0.33(8)
8.3689(11)	586.14(22)	175.09(5)	0.62(10)
8.3681(12)	585.99(24)	174.51(5)	0.71(10)
8.3696(12)	586.29(24)	173.95(5)	0.80(10)
8.3676(12)	585.87(25)	172.81(5)	0.99(11)
8.3623(12)	584.77(25)	171.58(5)	1.20(11)
8.3594(12)	584.15(26)	171.19(5)	1.27(11)
8.3595(12)	584.17(25)	170.78(5)	1.34(11)
8.3551(12)	583.25(26)	170.35(5)	1.42(11)
8.3512(12)	582.43(26)	169.79(5)	1.52(12)
8.3336(14)	578.76(28)	167.98(5)	1.86(13)
8.3306(14)	578.14(28)	167.53(5)	1.95(13)
8.3214(13)	576.22(28)	166.68(5)	2.13(13)
8.3091(14)	573.67(30)	165.60(5)	2.35(14)
8.2994(14)	571.67(28)	164.91(5)	2.50(13)
8.2941(14)	570.57(29)	164.28(5)	2.64(14)
8.2801(13)	567.68(27)	163.19(5)	2.88(14)
8.2660(14)	564.79(29)	161.94(5)	3.18(15)
8.2483(15)	561.16(31)	160.89(5)	3.44(15)
8.2333(18)	558.10(36)	159.97(5)	3.67(16)

References

(1) Arp, V. D.; McCarty, R. D.; Friend, D. G. *Thermophysical Properties of Helium-4 from 0.8 to 1500 K with Pressures to 2000 MPa*; NIST, United States Department of Commerce, 1998.

(2) Boehler, R.; Kennedy, G. C. Equation of State of Sodium Chloride up to 32 kbar and 500°C. *J. Phys. Chem. Solids* **1980,** *41*, 517.
